# Inflammation Promotes Proteolytic Processing of the Prohormone Chromogranin A by Macrophages

**DOI:** 10.1210/jendso/bvaf090

**Published:** 2025-05-15

**Authors:** Melina Ioannidis, Hendrik van Dijk, Elke M Muntjewerff, Venkat R Chirasani, Harry Warner, Willemijn van den Dool, Pieter Grijpstra, Frans Bianchi, Sushil K Mahata, Geert van den Bogaart

**Affiliations:** Department of Molecular Immunology, Groningen Biomolecular Sciences and Biotechnology Institute, University of Groningen, 9747 AG Groningen, the Netherlands; Department of Molecular Immunology, Groningen Biomolecular Sciences and Biotechnology Institute, University of Groningen, 9747 AG Groningen, the Netherlands; Department of Medical Cell Biology, Uppsala University, Uppsala 75237, Sweden; Department of Biochemistry & Biophysics, University of North Carolina School at Chapel Hill, Chapel Hill, NC 27599-7260, USA; Department of Molecular Immunology, Groningen Biomolecular Sciences and Biotechnology Institute, University of Groningen, 9747 AG Groningen, the Netherlands; Department of Molecular Immunology, Groningen Biomolecular Sciences and Biotechnology Institute, University of Groningen, 9747 AG Groningen, the Netherlands; Department of Molecular Immunology, Groningen Biomolecular Sciences and Biotechnology Institute, University of Groningen, 9747 AG Groningen, the Netherlands; Department of Molecular Immunology, Groningen Biomolecular Sciences and Biotechnology Institute, University of Groningen, 9747 AG Groningen, the Netherlands; VA San Diego Healthcare System, San Diego, CA 92161, USA; Department of Medicine, University of California San Diego, Stein Clinical Research Building, La Jolla, CA 92093-0732, USA; Department of Molecular Immunology, Groningen Biomolecular Sciences and Biotechnology Institute, University of Groningen, 9747 AG Groningen, the Netherlands; Department of Medical Biology and Pathology, University Medical Center Groningen, 9713 GZ Groningen, the Netherlands

**Keywords:** macrophages, chromogranin A, proteolytic processing, inflammation

## Abstract

Chromogranin A (CgA), a 439–amino acid–long protein produced by neuroendocrine cells, is critical in health and disease. Through proteolytic processing, CgA is transformed into several bioactive peptides. These peptides, as well as CgA, have been implicated in various pathological conditions. Interestingly, CgA-derived peptides have opposing effects, such as catestatin (CST) and pancreastatin (PST), which have contrasting immunomodulatory properties. PST promotes a proinflammatory response, increasing the production of proinflammatory cytokines, whereas CST reduces proinflammatory and increases anti-inflammatory cytokines in mice. However, how CgA and CgA-derived peptides regulate the immune response is unknown, and most of our knowledge is based on mouse studies. Since multiple studies suggest that CgA and CgA-derived peptides influence macrophages, we aimed to investigate the interaction between CgA and human monocyte-derived macrophages. Therefore, we tested whether human macrophages produce CgA, are affected by CST and PST, and/or produce CST and PST. We found that human monocyte-derived macrophages and other immune cells do not produce CgA, and CST and PST have only minor effects on cytokine production and immune metabolism. However, proteases involved in the cleavage of CgA are differentially expressed in macrophages depending on their inflammatory phenotype, suggesting that CgA is increasingly converted into CST and PST in inflammatory conditions. As levels of CgA and its cleavage products CST and PST are associated with human diseases, it is essential to understand how they influence the immune response.

Chromogranin A (CgA) is a prohormone belonging to the Granin family [1], which is secreted by neuroendocrine, endocrine, and enteroendocrine cells [2]. The primary intracellular function of CgA is to sort cargo proteins like insulin into secretory granules [3]. However, CgA also has extracellular functions regulating metabolism, the cardiovascular system, and the immune system [4-8]. Therefore, CgA-knockout (KO) mice (*Chga*^−/−^), which have a deletion of exon 1 and an approximately 1.5 kbp proximal promoter that completely inactivates the *Chga* allele, are obese [9], insulin sensitive [10], and hypertensive with higher plasma catecholamine levels [9]. Multiple studies have correlated elevated plasma levels of circulating CgA with pathological conditions, including neuroendocrine tumors [11-13], heart failure [14-16], renal failure [17, 18], hypertension [9, 14, 16], rheumatoid arthritis [19], and inflammatory bowel disease [20]. Moreover, low CgA correlates with high diversity in the gut microbiome [21]. This, combined with the fact that CgA is highly conserved between species [2], shows that CgA is clearly important for health and disease [22-24].

The human CgA gene *CHGA* encodes a 439–amino acid mature prohormone [25] characterized by a coiled-coil structure [26]. CgA is proteolytically processed into several bioactive peptide hormones, including vasostatin 1 (human (h)CgA_1-76_), vasostatin 2 (hCgA_1-113_) [27], WE14 (hCgA_324-337_) [28], serpinin (hCgA_411-436_) [29], catestatin (CST) (hCgA_352-372_) [30], and pancreastatin (PST) (hCgA_250-301_) [31]. These peptides exert comprehensive regulatory effects, affecting glucose [32, 33] and calcium homeostasis [27, 34]. The endocrine, cardiovascular, and immune systems are also influenced by CgA-derived peptides [24, 35, 36]. Intriguingly, several CgA-derived peptides counteract each other, particularly CST and PST, which exert opposite immunomodulatory effects [23, 24].

PST influences the immune system toward proinflammatory signaling [32, 37]. For example, intraperitoneal injection with human PST (hCgA_291-319_) in mice increased the expression of proinflammatory genes coding for prointerleukin (IL)-1β, tumor necrosis factor-α (TNF-α), monocyte chemoattractant protein (Mcp)-1, and inducible nitric oxide synthase (iNos) in white adipose tissue both of wild-type and *Chga*^−/−^ diet-induced obese mice [32]. Moreover, PST can promote the differentiation of murine peritoneal macrophages to a proinflammatory phenotype [37]. PST is also related to human disease, as increased plasma levels of PST are found in patients with neuroendocrine tumors [22, 24, 38], as well as in type 2 diabetes [39].

In contrast to PST, CST exerts anti-inflammatory effects. CST-KO mice, with 63 bp of the CST domain deleted from exon VII of the *Chga* gene, have increased expression of proinflammatory cytokines in the colon [5] and heart [7] compared to wild-type mice. In addition, intraperitoneal injection of exogenous CST to the CST-KO mice reversed this phenotype, increasing anti-inflammatory proteins [5, 7]. Human studies have shown that patients suffering from chronic hypertension have lower levels of circulating CST [9, 36, 40, 41], whereas plasma levels of CST are elevated in patients with inflammatory bowel disease [5]. In vitro, the treatment both of M1 (proinflammatory) and M2 (anti-inflammatory) bone marrow–derived macrophages (BMDMs) of wild-type mice with CST resulted in small but significant increases in the production of the anti-inflammatory cytokine IL-10 and a decrease of proinflammatory cytokines TNF-α, IL-1β, CCL-2, CCL-3, and CXCL-1 [7]. Moreover, the inflammatory phenotype of the CST-KO mouse is reversible by bone marrow transplantation and by depleting macrophages with chlodronate liposomes [7]. Based on these findings, the anti-inflammatory effects of CST have been attributed to macrophages [7, 38, 42].

Thus, the immunomodulatory effect of CST and possibly PST are at least partly due to their effect on immune cells, including macrophages and other bone marrow–derived cells. However, CgA and CST were both detected by Western blot in lysates of induced peritoneal macrophages of wild-type mice [7], suggesting that macrophages also produce CgA. Alternatively, or additionally, they might also be involved in the proteolytic processing of CgA into CST and PST, as they could potentially ingest CgA and modify it. CST and PST can be generated by several proteases, including prohormone convertases (PCs) 1/3 and 2 [43-45], cathepsin L [46], thrombin [47], and plasmin [48, 49]. While the specific cleavage sites of these proteases and their regulation remain unclear, PC1/3 and PC2 are well known for converting other prohormones, like proinsulin and proglucagon [50, 51]. Moreover, cathepsin L, an endosomal cysteine protease, is linked to pathological conditions such as atherosclerosis and type 2 diabetes [52]. Thrombin, an extracellular serine protease, is key in the blood coagulation cascade [53], while plasmin is a primary fibrinolytic enzyme [54].

Most of the knowledge about CST and PST in the immune system is from studies conducted in mice. As levels of CgA and its cleavage products CST and PST are associated with human disease, it is essential to understand if human macrophages (i) produce CgA, (ii) are affected by CST and PST, and (iii) convert CgA into CST and/or PST. Therefore, we addressed these questions in human peripheral blood monocyte–derived macrophages.

## Materials and Methods

### Generation of Human Monocyte-derived Macrophages

Buffy coats of healthy donors were obtained from the Dutch blood bank. The Dutch blood bank approved the use of human blood, and all experiments were conducted following national and institutional guidelines. The Dutch blood bank obtained informed consent. Samples were anonymized, and none of the investigators could determine the identity of the blood donors.

Peripheral blood mononuclear cells were isolated from buffy coats of healthy donors using a standard density gradient centrifugation with Lymphoprep (STEMCELL Technologies, 07861) as described earlier [55]. Monocytes were isolated from the peripheral blood mononuclear cell fraction by magnetic-activated cell sorting using CD14 microbeads (Miltenyi Biotec, 130-114-976). Isolated monocytes were cultured in ultra-low adherent 6-well plates (Corning, CLS3471-EA) for 7 days containing 2 mL of complete media (RPMI 1640) (Gibco, 11530586), 10% fetal bovine serum (FBS) (Hyclone, 10309433), 2 mM L-glutamine (Gibco, 15430614), 1% Antibiotic-Antimycotic (Gibco, 15240062) enriched with 100 ng/mL recombinant human macrophage colony-stimulating factor (M-CSF) protein (R&D Systems, 216-MC) at 37 °C and 5% CO_2_. The naive macrophages (M0) were treated with 100 ng/mL lipopolysaccharide (LPS) (Sigma-Aldrich, L2630) and 20 ng/mL interferon-γ (IFN-γ) (Peprotech, 300-02) or 300 U/mL IL-4 (Miltenyi Biotec, 130-093-924) for 48 hours toward a proinflammatory- (M1) or anti-inflammatory (M2) phenotype, respectively.

### Neutrophil Isolation

Leftover neutrophils from our previous research were isolated from heparinized venous blood through a two-step procedure as described previously [56]. First, blood was separated using Ficoll Histopaque (Sigma Aldrich) density gradient centrifugation, allowing neutrophil separation. The neutrophil layer was carefully collected and further purified by subjecting the cell suspension to hypotonic lysis, effectively removing erythrocyte contamination while preserving the integrity of neutrophils. Neutrophils were lysed and used to prepare complementary DNA (cDNA), as described next.

### Isolation and Generation of Mouse Bone-marrow-derived Macrophages

BMDMs were generated from female and male C57BL/6J mice. The tibia and femur were flushed out with BMDM media (RPMI 1640) (Gibco, 11530586), 10% FBS, 2 mM L-glutamine, 1% Antibiotic-Antimycotic, and 55 µM β-mercaptoethanol (Gibco, 31350010) to obtain bone marrow cells. Bone marrow cells were cultured at 2 to 4 × 10^6^ cells per Petri dish in 13 mL BMDM media containing 50 ng/mL recombinant murine M-CSF (Preprotech, 315-02) for 7 days. On day 7 of differentiation, naive (M0) macrophages were treated with 100 ng/mL LPS (Sigma-Aldrich, L2630) and IFN-γ (BD Bioscience, 554587) or IL-4 (Miltenyi, 130-094-061) for 48 hours to polarize the cells to a proinflammatory (M1) or anti-inflammatory (M2) phenotype, respectively.

### Isolation of Murine Pancreatic Islet Cells

As a positive control for the expression of murine *Chga*, pancreatic islet cells were isolated from C57BL/6J mice as described earlier [57]. Briefly, the pancreas of euthanized mice was injected with 2.5 mg/mL of Collagenase A (Roche, 10103578001) in Hank's Balanced Salt Solution (HBSS) (Gibco, 24020117) and incubated for 18 minutes at 37 °C. Subsequently, the pancreas was centrifuged in Rinse Buffer (HBSS enriched with penicillin-streptomycin and 2.5 mM glucose) and HistoPaque (Sigma-Aldrich, HISTOPAQUE-1077) to separate the pancreatic islets cells from the exocrine tissue.

### Cell Line

HEK293 (Research Resource Identifier RRID:CVCL_0045) is a human epithelial cell line that was cultured in complete media containing Dulbecco’s modified Eagle’s medium (Gibco, 11500416), 10% FBS, 2 mM L-glutamine, 1% Antibiotic-Antimycotic at 37 °C, and 5% CO_2_. HEK cells were tested for mycoplasma every 3 months using a MycoAlert Mycoplasma Detection Kit (Lonza, LT07-318).

### Jet-PEI Transfection of HEK293 Cells With CHGA-eGFP Construct

For the generation of a positive control to measure the expression of human *CHGA* by quantitative polymerase chain reaction (qPCR), the CHGA_OHu20175C_pcDNA3.1(+)-C-eGFP construct (synthetic construct by GenScript; deposited at Addgene, 228569) was introduced in the HEK293 cell line using jetPEI (Polyplus-Transfection, 101000053) using manufacturer guidelines. Briefly, for the transfection approximately 50 000 to 100 000 HEK293 cells were plated in a 24-well plate. For the transfection, 2 µg of DNA and 4 µL jetPEI were diluted in 150 mM NaCl before being combined and added to the cell suspension. Cells were incubated with the construct overnight at 37 °C and 5% CO_2_.

### Confirmation of HEK293 Transfection Using Confocal Microscopy

Expression of green fluorescent protein (GFP)-tagged CgA in the transfected HEK293 cells was checked by microscopy. Around 50 000 transfected and non-transfected HEK293 cells were seeded on coverslips and fixed using 4% paraformaldehyde (PFA) (Aurion, 15710) for 15 minutes at 4 °C. The cells were washed 3 times with phosphate-buffered saline (PBS) before being blocked and permeabilized with CLSM buffer (PBS + 20 nM glycine + 3% bovine serum albumin) and 0.1% saponin (Sigma, 47036-50G-F) for 30 minutes at 4 °C. After blocking, the samples were incubated with rabbit-anti-GFP (1:100; Research Resource Identifier [RRID]:AB_305564) for 1 hour at room temperature. Samples were washed 3 times with CLSM + 0.1% Saponin before incubating with Phalloidin-647 (1:200; ThermoFisher, A22287) and Goat-anti-rabbit-488 (1:200; RRID:AB_2576217) for 1 hour at room temperature. Subsequently, the cells were incubated with 4′,6-diamidino-2-phenylindole dihydrochloride (DAPI) (1:3000; Sigma Aldrich, 32670) for 10 minutes followed by 3 washes with distilled water before mounting samples on glass slides with Prolong Diamond (Invitrogen, P36961). Samples were imaged using an LSM800 Airyscan microscope with a 63× (N/A 1.4) oil lens. Images were analyzed using FIJI just ImageJ software.

### RNA Isolation, Complementary DNA Synthesis, and Reverse-Transcription Quantitative Polymerase Chain Reaction

Reverse-transcription polymerase chain reaction (RT-PCR) was performed to determine the expression levels of *CHGA/Chga* and proteases in different cell types. RNA was isolated using the Quick-RNA MiniPrep kit (ZymoResearch, R1054) in accordance with the manufacturer's instructions. Subsequently, the Moloney murine leukemia virus RT (M-MLV-RT) kit (Invitrogen, 28025-021) was used to generate cDNA following the manufacturer's instructions. The cDNA was used to perform RT-qPCR using 5 µL of the Sybr Green Master Mix (Applied Biosystems, A25742), and the primers used are listed in Table 1.

**Table 1.** Primers

Gene name	Species	Primer sequence	Company
*SNRPD3*	*Homo sapiens* (housekeeping)	Fwd 5′GGAAGCTCATTGAAGCAGAGGAC′3 Rv 5′CAGAAAGCGGATTTTGCTGCCAC′3	Sigma
*CHGA*	*Homo sapiens*	Fwd 5′TAAAGGGGATACCGAGGTGATG′3 Rv 5′TCGGAGTGTCTCAAAACATTCC′3	Sigma
*Chga*	*Mus musculus*	Fwd 5′AGAACCAGAGCCCTGATGCCAA′3 Rv 5′CTCTGTGGTTGCCTCAAAGCCA′3	Sigma
*Actb*	*Mus musculus* (housekeeping)	Fwd 5′CATTGCTGACAGGATGCAGAAGG′3 Rv 5′TGCTGGAAGGTGGACAGTGAGG′3	Sigma
*CTSL*	*Homo sapiens*	Fwd 5′GAAAGGCTACGTGACTCCTGTG′3 Rv 5′CCAGATTCTGCTCACTCAGTGAG′3	Sigma
*PCSK1*	*Homo sapiens*	Fwd 5′CCAGATGTGCAGGAGAAATTGCC′3 Rv 5′CCGTCACAATGCCATCCAGCAT′3	Sigma

The qPCR program was set as follows: 50 °C for 2 minutes, 95 °C for 2 minutes, 95 °C for 15 seconds, and 60 °C for 1 minute. The program was repeated 40 times, and the results were analyzed using the 2^−ΔΔCt^ method as described earlier [58].

### Measurement of Cytokine Production by Human Monocyte-derived Macrophages

Human monocyte–derived macrophages were seeded at 30 000 cells per well in a flat-bottom plate and stimulated 200 nM human PST (PEGKGEQEHSQQKEEEEEMAVVPQGLFRG-amide, CgA_273-301_) or 1 µM human CST (SSMKLSFRARAYGFRGPGPQL, CgA_352-372_) in the presence and absence of 100 ng/mL LPS (Sigma-Aldrich, L2630). Cytokine production was quantified using enzyme-linked immunosorbent assays for TNF-α (RRID: AB_2575097) and IL-6 (RRID: AB_2574995) according to the manufacturer's protocol.

### Cyclooxygenase-2 Expression and Metabolic Changes

For the internal cyclooxygenase-2 (COX-2) staining and metabolic probe treatment, 2 × 10^6^ isolated human CD14^+^ monocytes were cultured in ultra-low adherent 6-well plates (Corning, CLS3471-EA) for 7 days containing 2 mL of complete media (RPMI 1640, 10% FBS, 2 mM L-glutamine, 1% Antibiotic-Antimycotic) containing 100 ng/mL recombinant human M-CSF protein and 200 nM human PST or 1 µM human CST at 37 °C and 5% CO_2_.

For the internal COX-2 staining, 100 000 cells per well were seeded in a 96-V-bottom plate and stained with Fixable Viability Dye eFluor780 (eBioscience, 65-0865-14, 1:1000 in cold PBS) for 15 minutes on ice prior to fixation with 4% PFA for 15 minutes at 4 °C. Cells were washed several times in cold PBS and stored overnight at 4 °C. The next day, cells were blocked and permeabilized in PBS containing 2% human serum and 0.05% saponin for 30 minutes at 4 °C. Subsequently, cells were incubated with an anti-human-COX-2 (PE-labeled) (RRID: AB_2739077) for 30 minutes at room temperature. Finally, cells were washed twice in PBS before the samples were analyzed using the CytoFlex S (Beckman Coulter).

Metabolic changes were analyzed using several probes. Therefore, 100 000 macrophages per well were seeded in a 96-well ultra-low adherence plate (Corning, 10023683) and incubated with 3.5 µM BODIPY 493/503 (lipid storage compartments), 1 µM BODIPY FL C12 (fluorescent fatty acid analogue), or 50 µM 2-NBDG (fluorescent glucose analogue) probes for 15 minutes at 37 °C and 5% CO_2_. Subsequently, cells were washed in phenol red-free RPMI 1640 (Gibco, 11835030), collected, and resuspended in a V-bottom 96-well plate and analyzed using the CytoFlex S (Beckman Coulter).

### Determination of Intracellular Lactate Levels

Approximately 10 000 cells/well were seeded in a 96-well flat-bottom plate (Corning, 3596) and treated with PST (200 nM) or CST (1 μM) in the presence or absence of LPS (100 ng/mL) for different time points (0 hours, 3 hours, 6 hours, 24 hours). Intracellular lactate levels were measured using the Lactate-Glo assay (Promega, J5021) following manufacturer guidelines. Lactate was measured as luminescence using the Lactate-Glo assay, which couples lactate oxidation to NADH production, driving a bioluminescent reaction in which NADH converts a proluciferin substrate into luciferin, generating light via luciferase, which was measured using a Synergy Mx reader (BioTek).

### Succinate Dehydrogenase Activity (Complex II)

Around 10 000 cells/well were seeded in a 96-well flat-bottom plate (Corning, 3596) and treated with PST (200 nM) or CST (1 μM) in the presence or absence of LPS (100 ng/mL) for different time points (0 hours, 3 hours, 6 hours, 24 hours). Succinate dehydrogenase (SDH) activity was measured using the 3-(4,5-dimethyl-2-thiazolyl)-2,5-diphenyl-2H-tetrazolium bromide (MTT) (Sigma Aldrich, M2128) assay. Following treatment, cells were exposed to a 0.5 mg/mL solution of MTT (Sigma Aldrich, M2128) and incubated for 3 hours at 37 °C. After incubation, the cells were rinsed once with PBS and then treated with DMSO for 10 minutes at 37 °C. Absorbance was subsequently recorded at 540 nm using a Synergy HTX multimode reader (BioTek).

### Uptake of Catestatin–Fluorescein Isothiocyanate by Human Macrophages

A total of 50 000 monocyte-derived macrophages were seeded on poly-L-lysine coated cover glasses. Cells were incubated without or with different concentrations (1 μM and 10 μM) CST–fluorescein isothiocyanate (FITC) (GenScript) for 2 hours at 37 °C and fixed using 4% PFA for 15 minutes at 4 °C. Subsequently, cells were blocked and permeabilized with CLSM buffer and 0.1% saponin for 30 minutes at 4 °C. Afterward, cells were stained with mouse IgG1 ascites directed against FITC (RRID: AB_3695987) before staining with secondary Alexa Fluor 488–labeled secondary antibody directed against mouse immunoglobulin G (RRID:AB_141607) and Alexa Fluor 546-labeled phalloidin (Invitrogen, A22283). Samples were mounted with DAPI and imaged using an LSM800 microscope with a 63× (N/A 1.4) oil lens. Images were analyzed using FIJI just ImageJ software.

For flow cytometry, cells were seeded in a 96-well U-bottom plate at a density of approximately 100 000 cells/well and treated with 10 μM FITC, FITC dextran, or CST-FITC for 2 hours at 37 °C. The amount of FITC was verified using the NanodropOne (ThermoFisher). Cells were measured using the CytoFlex S (Beckman Coulter), and data were analyzed using NovoExpress software (Agilent).

### Processing Chromogranin A by Macrophages Using Förster Resonance Energy Transfer–based Biosensors

Four synthetic DNA constructs were generated by GenScript as follows: N-terminal targeting sequence of residues 1 to 115 of human CgA, mCitrine donor fluorophore, N or C-terminal cleavage site of CST or PST, and mScarlet-I acceptor fluorophore. The 10 amino acid long linkers linkers carry the cleavage sites of human CgA that are responsible for the generation of CST or PST with 5 amino acids downstream and 5 amino acids upstream of each cleavage site: i) PST-N _245-_KEIRK GESRS_-254_ (Addgene, 228571), ii) PST-C _297-_GLFRG GKSGE_-306_ (Addgene, 228570), iii) CST-N _347-_EDNRD SSMKL_-356_ (Addgene, 228573), iv) CST-C _368-_PGPQL RRGWR_-377_ (Addgene, 228571). The backbone of the plasmids is pcDNA3.1(+). Plasmids have been deposited at Addgene.

Using the Neon transfection system (Fisher Scientific, 10090314), macrophages were transfected with the Förster resonance energy transfer (FRET)-based biosensors. The 10 μL tip protocol (1000 V, 40 ms, 2 pulses) was used using 1 μg plasmid per transfection of 1.0 × 10^6^ cells in T buffer. Cells were transferred to a 4-chamber microscopy plate (Greiner, 627870) with warm phenol red free RPMI (Gibco, 32404014), supplemented with L-glutamine and Antibiotic-Antimycotic, and incubated for 1 hour at 37 °C before adding 10% FBS and 100 ng/µL M-CSF. The cells were incubated overnight at 37 °C and 5% CO_2_. The next day, cells were imaged directly or after fixation with 4% PFA, using a home-built TIRF microscopy as described previously [59]. Briefly, the samples were excited using a 488-nm Sapphire Laser System (Coherent, SKU 040082). Both green and red emissions were measured separately using an image splitter. The images were then analyzed in FIJI just ImageJ software using macros (Supplementary information: ImageJ scripts [60]). The FRET efficiency is defined as the ratio of the acceptor fluorescence (ie, sensitized emission) over the donor fluorescence.

### Statistical Analysis

Statistical analyses were performed using GraphPad software. Normality was tested using the Shapiro-Wilk test. Statistical tests are indicated in the figure legends. A *P* value less than .05 was considered statistically significant.

## Results

### Chromogranin A Is Expressed in Murine Bone Marrow but not in Murine and Human Macrophages

**Figure 1.**
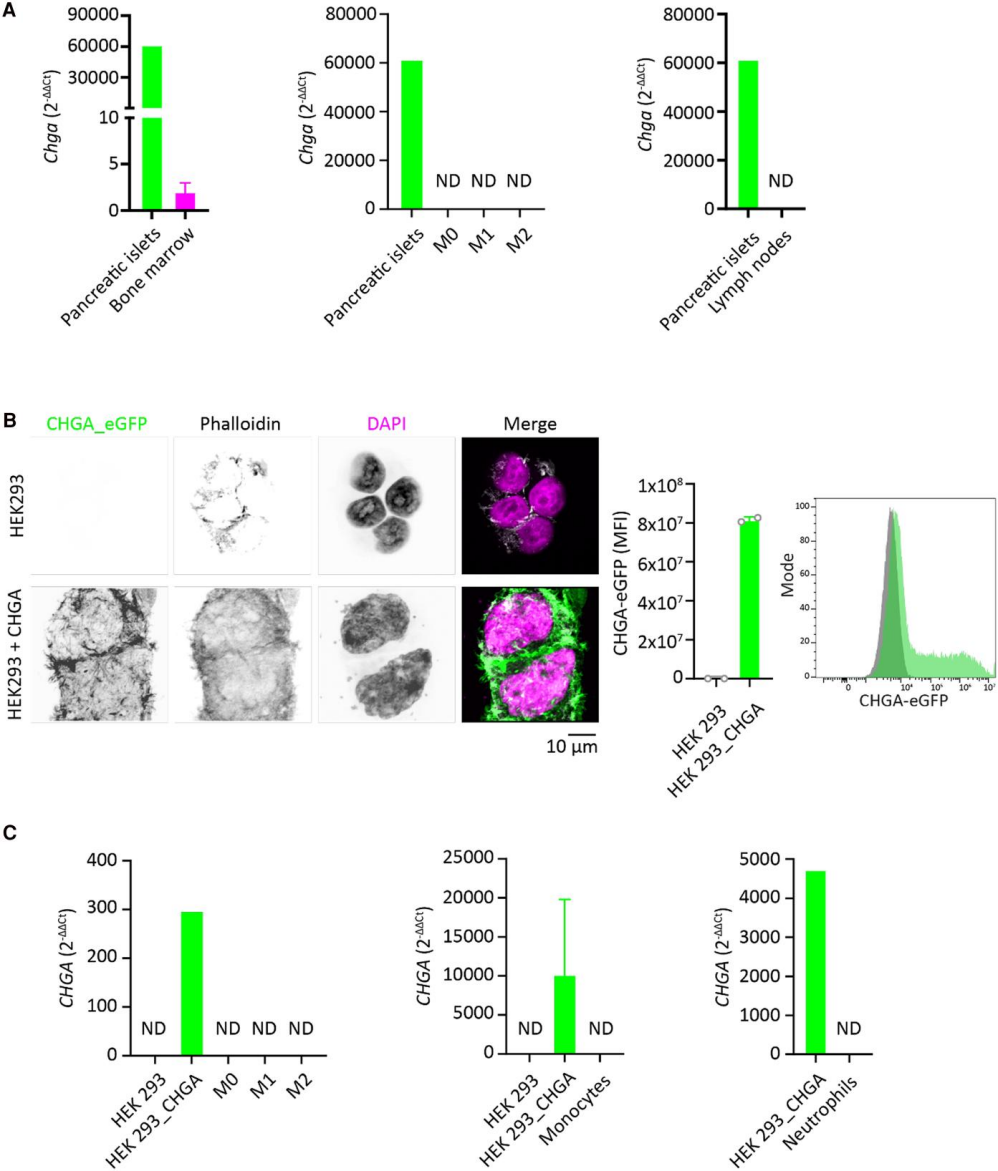
Expression of *Chga*/*CHGA* in mouse and human immune cells. A, *Chga* expression in bone marrow (n = 3), bone marrow-derived naive (M0) (n = 3), proinflammatory (M1; LPS + IFN-γ), and anti-inflammatory (M2; interleukin-4 [IL-4]) (n = 4) macrophages and lymph nodes (n = 2) of wild-type C57BL/6 mice. Pancreatic islet cells were used as a positive control. B, As a positive control for human *CHGA* expression, HEK293 cells were transfected with a construct coding for GFP-tagged chromogranin A (CgA). Results of transfection are validated by microscopy (left) and flow cytometry (right). Each data point represents one experiment (n = 2). C, *CHGA* expression in naive (M0), proinflammatory (M1; LPS and IFN-γ), and anti-inflammatory (M2; IL-4) human monocyte-derived macrophages (n = 4), neutrophils (n = 3), and monocytes (n = 3). Averages shown; error bars display the SEM.

Abbreviation: ND, not detected.

*CHGA/Chga* gene expression in human and murine immune cells was investigated using RT-qPCR (Fig. 1A). *Chga* was expressed in murine bone marrow, albeit at much lower levels than in pancreatic islets known to express high levels of *Chga* [61]. Nevertheless, because the total volume of bone marrow is far greater than that of pancreatic islets, bone marrow could still be a major source of CgA. However, *Chga* expression was not detected in cultured murine BMDMs or isolated murine lymph nodes (Fig. 1A).

For human macrophages, we tested *CHGA* expression in peripheral blood–derived macrophages, peripheral blood CD14^+^ monocytes (ie, precursors of macrophages), and peripheral blood neutrophils. As a negative control, we used HEK293 cells. As a positive control, we transfected HEK293 cells with a plasmid carrying the *CHGA* gene genetically fused to GFP (Fig. 1B). Our data show that *CHGA* is not expressed in human monocytes, neutrophils, and monocyte-derived macrophages regardless of their polarization toward a proinflammatory (M1; LPS and IFN-γ) or anti-inflammatory (M2; IL-4) phenotype (Fig. 1C).

### Pancreastatin and Catestatin Influence Interleukin-6 and Cyclooxygenase-2 Production in Human Macrophages

To investigate whether PST and CST affect human macrophages, monocyte-derived macrophages were treated with LPS (100 ng/mL) in the presence or absence of PST (200 nM) or CST (1 μM). These concentrations are considerably higher than circulating concentrations in a nondisease state of 0.03 to 1.5 nM for CST [62], 0.5 to 5 nM for CgA [62], and 4 to 20 pM for PST [63, 64]. We used higher concentrations in this study because plasma levels of CgA and its derived peptides can increase substantially in disease conditions, and our concentrations reflect those observed in the context of inflammation [22, 38]. Moreover, this allowed for comparison with previous studies in which similar concentrations were used [32, 42]. These concentrations of CST and PST did not affect the viability of the cells (Supplementary Fig. S1 [60]).

**Figure 2.**
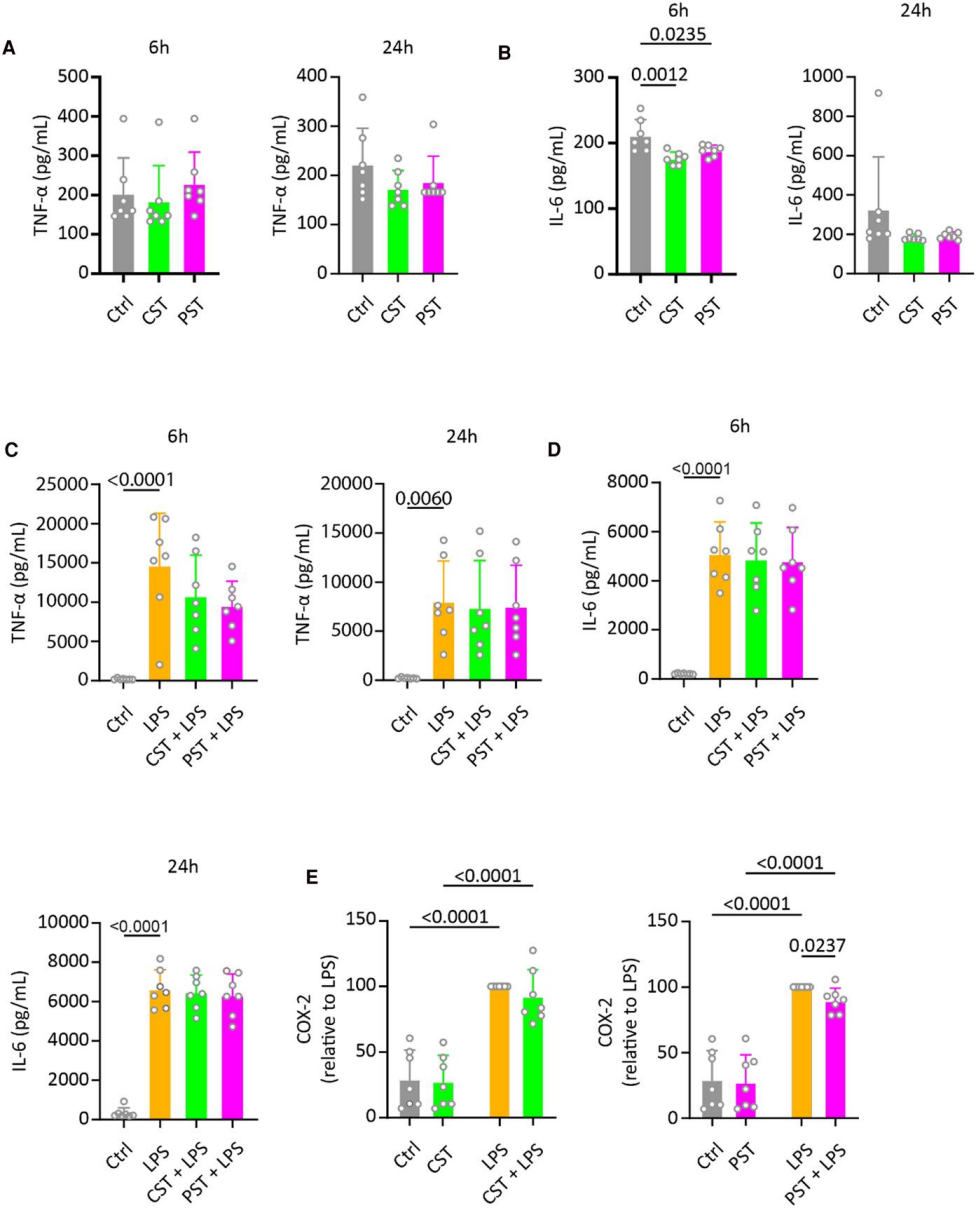
Catestatin (CST) and pancreastatin (PST) inhibit interleukin-6 (IL-6) production; PST also reduces COX2 production. A, Tumor necrosis factor α (TNF-α) and B, IL-6 production of human monocyte-derived macrophages measured after 6 hours’ and 24 hours’ treatment with CST (1 μM) or PST (200 nM). C, TNF-α and D, IL-6 production as in panels A and B, but now in the presence of lipopolysaccharide (LPS) (100 ng/mL). Each data point represents one donor (n = 7); error bars display the SEM. Data were analyzed with 1-way analysis of variance (ANOVA); *P* value is displayed in case of statistical significance (*P* < .05). E, COX-2 expression after treatment with CST or PST in the presence or absence of LPS. Each data point represents one donor (n = 7); error bars display the SEM. Data were analyzed with 2-way ANOVA. *P* value is displayed in case of statistical significance (*P* < .05).

Before use, PST and CST were checked by mass spectrometry (Supplementary Fig. S2 and Supplementary Table S1 [60]). After 6 hours’ and 24 hours’ treatment, the TNF-α and IL-6 levels were measured in the supernatant. Whereas CST and PST did not affect TNF-α production in the absence of LPS (Fig. 2A), IL-6 production was significantly reduced by both compounds after 6 hours’ incubation but not after 24 hours of treatment (Fig. 2B). In LPS-treated macrophages, neither PST nor CST affected the production of TNF-α and IL-6 (Fig. 2C). Lower concentrations of CST and PST also did not significantly affect cytokine production, regardless of the presence or absence of LPS (Supplementary Fig. S3A and S3B [60]). The variation among donors in cytokine production was very large (>2 orders of magnitude), as is well known for human immune cells [65].

Macrophages not only produce cytokines but are also a major source of prostaglandins through the conversion of arachidonic acid by COX2 [66]. Since these signaling molecules have important immunomodulatory functions, we tested the effect of CST and PST on COX2 expression in human monocyte–derived macrophages. CST did not significantly affect COX2 expression (Fig. 2E). However, PST showed a small but statistically significant decrease in COX2 expression compared to cells stimulated only with LPS (see Fig. 2E). Thus, our findings suggest that both CST and PST reduce IL-6 production, and PST also reduces COX2, although the effects are only small.

### Catestatin Promotes Lipid Storage by Human Macrophages

Macrophages are highly plastic and can change their phenotype depending on environmental factors. Metabolic switches accompany these changes in phenotype [67]. To carry out their effector function, such as phagocytosis, pathogen killing, and cytokine production, proinflammatory M1 macrophages enhance their glycolysis, increase their fatty acid synthesis, and shorten their Krebs cycle, which results in a reduction in oxidative phosphorylation by mitochondria and increased lactate production [68, 69]. In contrast, anti-inflammatory M2-like macrophages have decreased glycolysis and increased oxidative phosphorylation [70]. Metabolic switches are also seen in cultured hepatocytes [33] and adipocytes [71] after treatment with PST or CST. Therefore, the effect of PST and CST on the metabolism of human monocyte–derived macrophages was tested using different fluorescent probes and by assessing mitochondrial function via complex II and lactate production.

**Figure 3.**
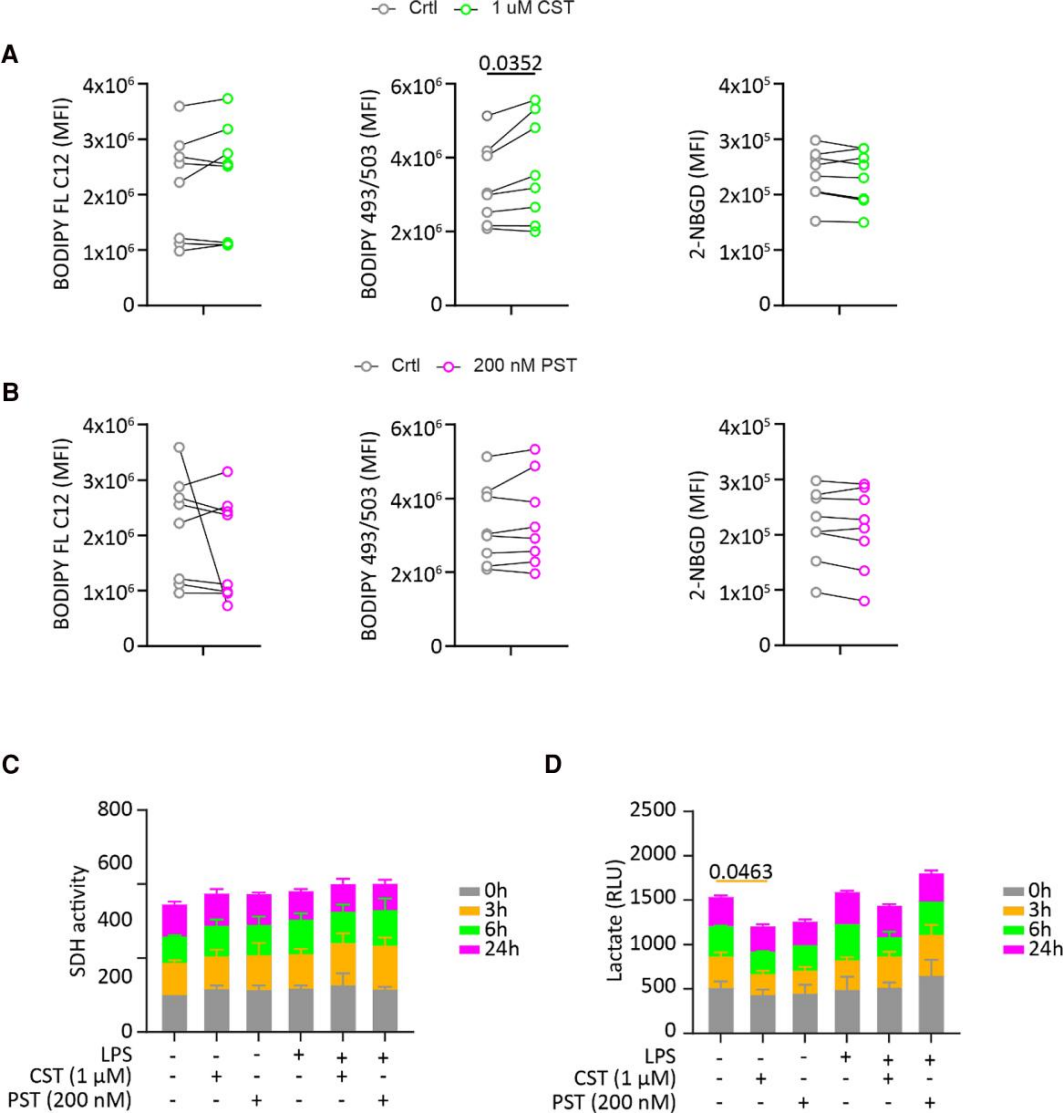
Catestatin (CST) promotes fat storage and lowers lactate production in human macrophages. Macrophages were treated with A, CST (1 μM) and B, pancreastatin (PST) (200 nM). Fat uptake was measured using BODIPY-FL C12. Fat storage was determined using BODIPY 493/53. Glucose uptake was inferred with 2-NBGD. Data display the mean fluorescence intensity. Each connected pair of data points represents one donor; data were analyzed with paired *t* tests, and the value displays the *P* value if statistically significant (*P* < .05). C, The effect of PST (200 nM) and CST (1 μM) on SDH activity (complex II) in both LPS-treated (100 ng/mL) and untreated macrophages at the indicated time points. The bar graph represents the mean ± SEM for n = 3 donors, with data normalized to the untreated condition at 0 hours. D, The effect of PST (200 nM) and CST (1 μM) on intracellular lactate levels in the presence and absence of lipopolysaccharide (LPS) (100 ng/mL) at the indicated time points. Data are presented as mean ± SEM. For C and D, 2-way analysis of variance was used for statistical analysis. The *P* value is displayed in case of statistical significance.

Human monocytes were differentiated into macrophages and treated with PST (200 nM) or CST (1 μM). The BODIPY FL C12 probe is a fat analogue and indicates changes in fat uptake. Our findings show that CST and PST did not affect BODIPY FL C12 uptake (Fig. 3A and 3B). However, the lipid storage probe BODIPY 493/503 signal was significantly increased in the presence of CST (see Fig. 3A) but not when treated with PST (see Fig. 3B). Moreover, glucose uptake was measured using 2-NBGD. There were no statistically significant changes in the presence of CST (see Fig. 3A) or PST (see Fig. 3B). CST and PST did not significantly alter SDH activity (Fig. 3C). However, CST significantly reduced lactate production in unstimulated macrophages, whereas PST had no effect (Fig. 3D).

### Macrophages Engulf Catestatin–Fluorescein Isothiocyanate and Express Proteases Involved in Chromogranin A Processing

**Figure 4.**
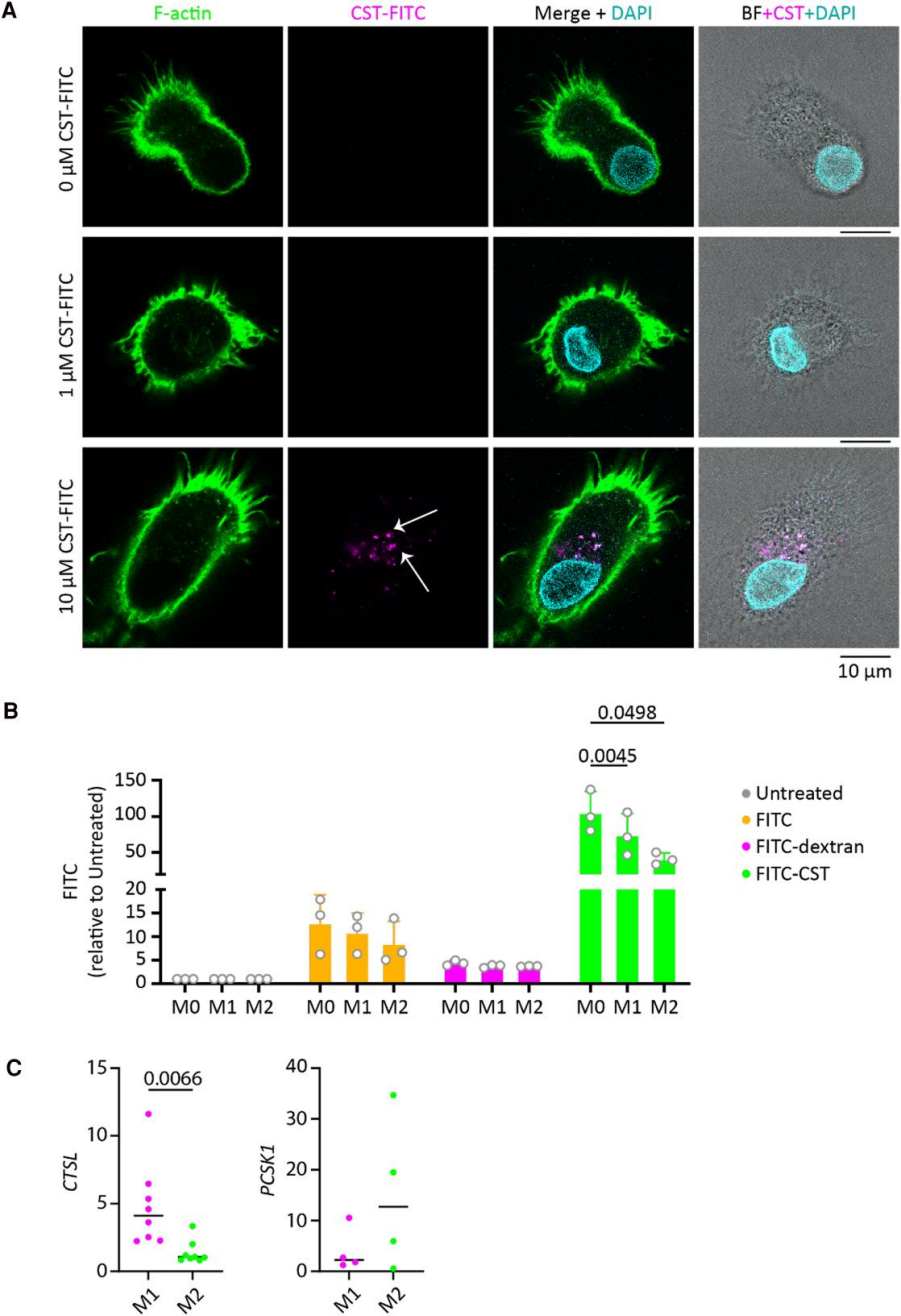
Macrophages ingest catestatin-fluorescein isothiocyanate (CST-FITC) and differentially express proteases involved in chromogranin A (CgA) processing. FITC-labeled CST was used to determine if human macrophages ingest CgA-derived peptides. A, Confocal microscopy images of macrophages treated with 1 µM or 10 µM CST-FITC (magenta), and labeled with phalloidin (green) and DAPI (4′,6-diamidino-2-phenylindole; blue). Scale bars, 10 μm. B, Uptake of FITC, FITC-dextran, and FITC-CST in naive (M0), proinflammatory (M1; lipopolysaccharide [LPS] + interferon-γ [IFN-γ]), and anti-inflammatory (M2; interleukin-4 [IL-4]) macrophages by flow cytometry. Each data point represents one donor (n = 3). Statistical significance was assessed using a 2-way analysis of variance. C, Expression by reverse-transcription quantitative polymerase chain reaction of genes coding for PC1/3 and cathepsin L. Human monocyte-derived macrophages were stimulated with either LPS and IFN-γ (M1) or IL-4 (M2). Each data point represents one donor, a paired *t* test was performed, and the value displays the *P* value in case of statistical significance (*P* < .05).

Macrophages are phagocytically and endocytically highly active [72]. We therefore tested whether macrophages could internalize exogenous CgA. Macrophages were treated with 1 μM and 10 μM of fluorescently labeled CST. Confocal microscopy revealed that macrophages efficiently engulf the CST-FITC in a dose-dependent manner (Fig. 4A). The CST-FITC was mainly localized in intracellular compartments. Unfortunately, we did not have FITC-labeled PST, so we could not test for PST uptake. Uptake was specific, as CST-FITC was more efficiently internalized than unconjugated FITC or FITC-dextran (Fig. 4B). In these experiments, we adjusted the CST-FITC, FITC, and FITC-dextran concentrations based on the absorbance of the FITC fluorophore (Supplementary Fig. S4 [60]). Moreover, naive macrophages (M0) more efficiently ingested CST-FITC than differentiated M1 or M2 macrophages (see Fig. 4B).

CgA can be proteolytically processed by the intracellular proteases PC1/3 [43-45] and cathepsin L [46-48]. Therefore, we tested whether macrophages expressed PC1/3 and cathepsin L and whether the expression depends on inflammatory activation. Our data show that anti-inflammatory macrophages (differentiated with IL-4) have significantly higher expression of cathepsin L compared to proinflammatory macrophages (IFN-γ and LPS), whereas expression of PC1/3 was not significantly different (Fig. 4C).

### Förster Resonance Energy Transfer–based Biosensors Visualize Chromogranin A Cleavage at Specific Sites

**Figure 5.**
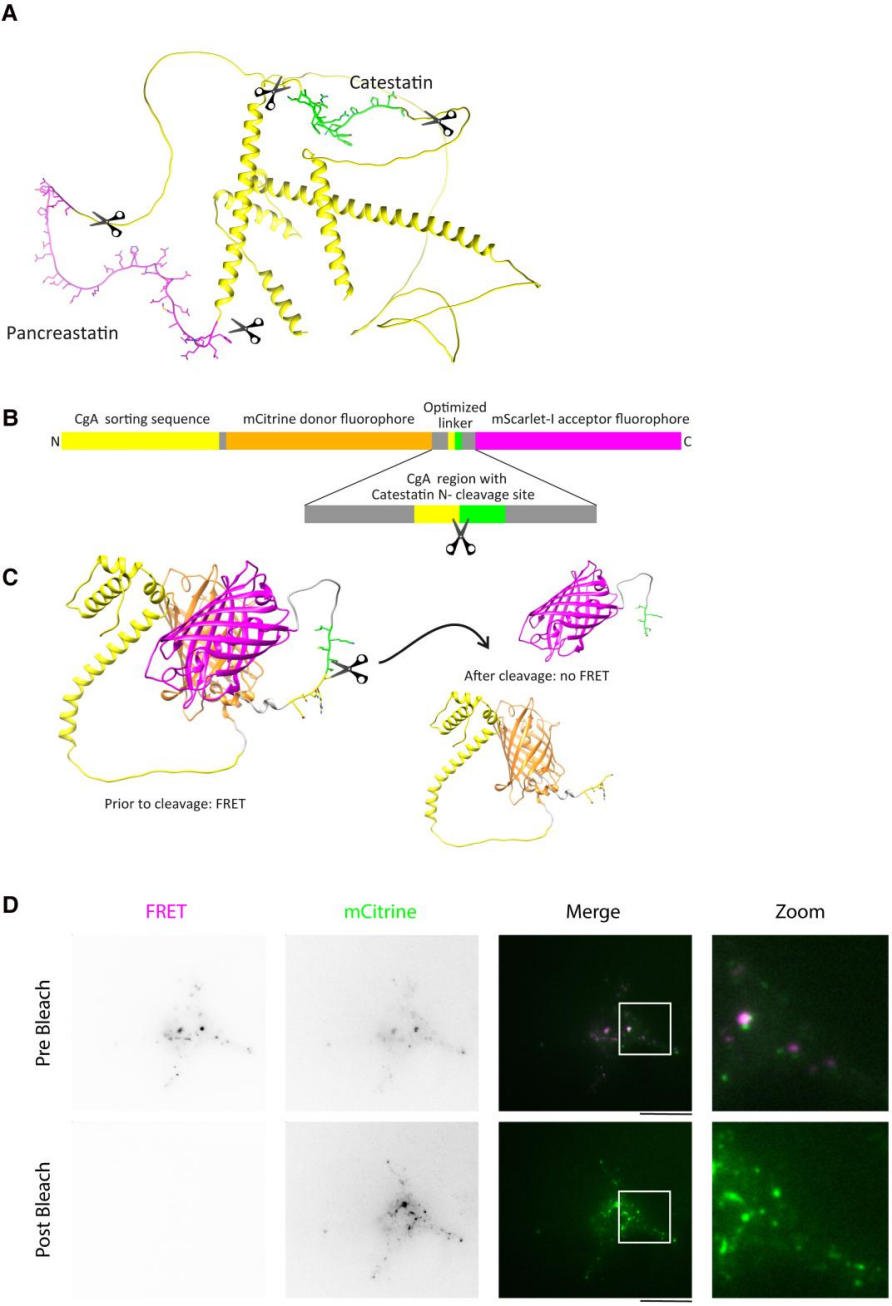
Förster resonance energy transfer (FRET) construct to determine proteolytic processing ofchromogranin A (CgA). A, AlphaFold-predicted structure of CgA with the pancreastatin (PST) andcatestatin (CST) regions indicated in pink and green, respectively. To determine CgA processing, 4 FRET-based probes were developed to detect proteolysis cleavage of CgA at position 351 (CST-N), 373 (CST-C), 249 (PST-N), and 301 (PST-C). B, Topology of CST-N FRET–based probe. C, The AlphaFold model shows the linker in an unstructured region. Proteolysis results in loss of FRET. D, Confirmation of FRET using acceptor photobleaching. The donor (mCitrine) was excited for 10 seconds at high light intensity. Due to donor protection, the acceptor was photobleached, whereas the donor fluorescence increased. Scale bars: 6.5 μm; white squares indicate the zoomed region.

Our findings support the hypothesis that macrophage inflammatory activation influences the proteolytic cleavage of CgA. However, since the precise cleavage sites of the PC1/3 and cathepsin L proteases on CgA are unknown, it remains unclear how this would affect the production of PST and CST. This question is difficult to resolve with existing assays because of low sensitivity and/or because assays cannot distinguish peptide fragments from full-length and/or incompletely processed CgA. Based on the CgA amino acid sequence, we predicted the protein structure using AlphaFold (Fig. 5A; see Supplementary Fig. S5 for the confidence of the predictions [60]) [73]. Both CST and PST are predicted to be located in unstructured loop regions of the protein, suggesting that the protease cleavage sites responsible for their generation are easily accessible to proteases. To investigate under which conditions this proteolytic processing occurs, we generated FRET-based probes to directly visualize proteolytic processing of discrete CgA cleavage sites.

The FRET probes contain a CgA sorting sequence, a donor fluorescent protein (mCitrine), which is connected to an acceptor fluorescent protein (mScarlet-I) by an unstructured linker peptide that carries one of the CgA cleavage sites responsible for the generation of CST and PST (Fig. 5B). Thereby, a total of 4 different FRET probes were generated carrying linker regions containing the N- or C-terminal cleavage sites flanging CST or PST. We believe this approach is justified because the AlphaFold modeling suggested that all cleavage sites for CST and PST are located in natively unstructured regions of CgA. As a sorting sequence, we used residues 1 to 115 of CgA, as previous work has shown that this region is required for correct intracellular targeting of CgA [74, 75]. Moreover, we selected mCitrine and mScarlett-I as donor and acceptor fluorophores because these have good optical properties and are pH stable (a prerequisite in acidic organelles). With our probes, the cleavage of the linker results in a loss of FRET signal (Fig. 5C).

To validate our probes, we first confirmed FRET using a principle known as donor photobleaching FRET. This principle is based on protecting the donor fluorophore against photobleaching (ie, mCitrine), because the FRET reduces the fluorescence lifetime of the donor molecule. The excitation of the donor fluorophore thus results in photobleaching of the acceptor (ie, mScarlet-I) instead of the donor fluorophore (ie, mCitrine), which in turn results in a reduction of the acceptor signal and an increase of the donor signal [76]. Indeed, we found that after 10 seconds of photobleaching the donor fluorophore with high light intensity, the fluorescence of the mCitrine channel markedly increased, whereas the signal for the mScarlet-I channel was reduced (Fig. 5D), confirming that the constructs report FRET.

**Figure 6.**
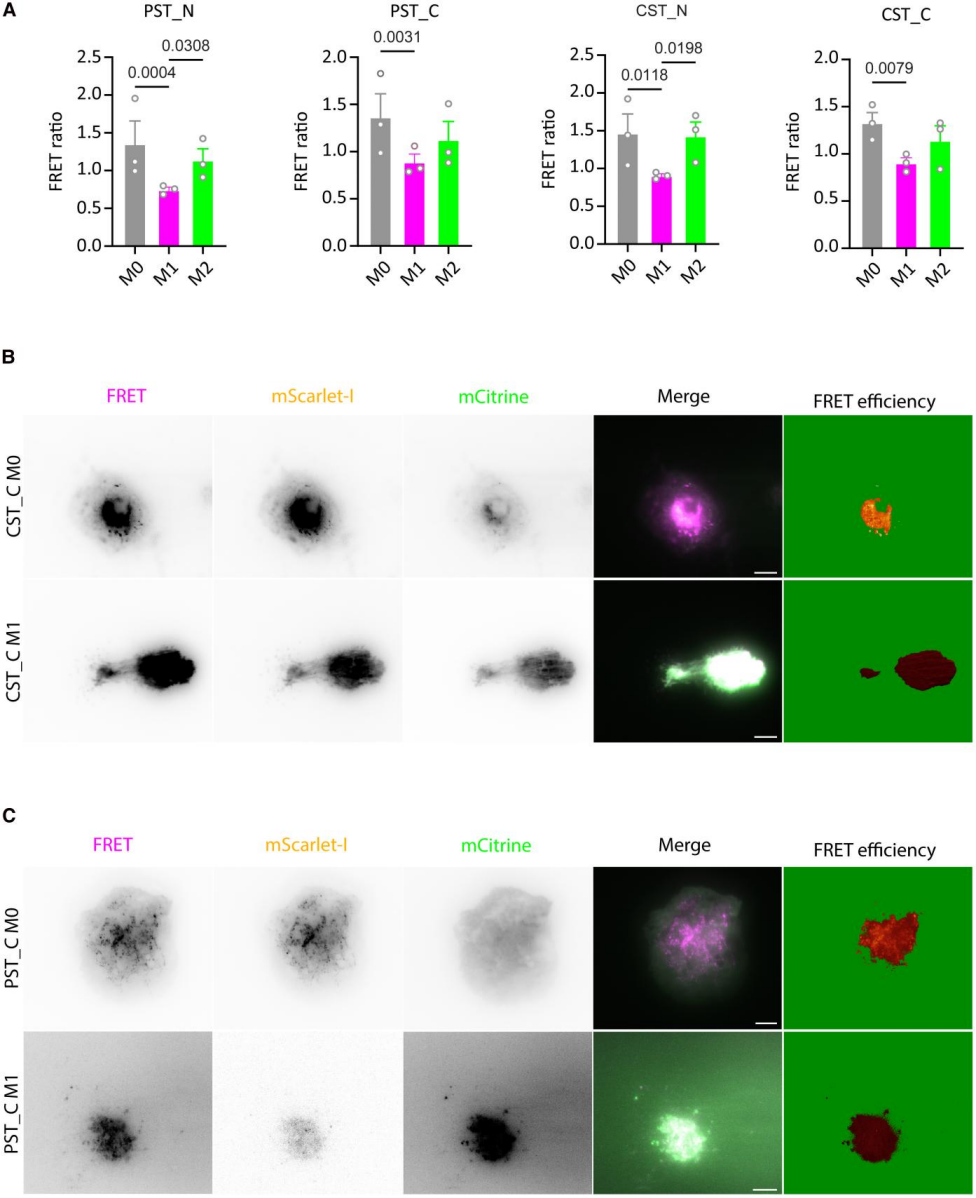
Inflammation promotes proteolytic processing of chromogranin A (CgA) by macrophages. Four different Förster resonance energy transfer (FRET) constructs were used to test the ability of human monocyte-derived macrophages to cleave CgA at the N-terminal and C-terminal of the pancreastatin (PST) and catestatin (CST) regions. A, FRET ratios, defined as the sensitized emission over the donor signal, show that proinflammatory macrophages (M1) have a higher efficiency of cleavage of the FRET constructs compared to nonactivated (M0) and anti-inflammatory (M2) macrophages. Each data point represents the mean of each donor (>5 cells/donor). The data are displayed as SEM; if statistically significant, the *P* value is indicated (*P* < .05). Data were analyzed using 2-way analysis of variance. B and C, FRET signal of B, CST C-terminal and C, PST C-terminal construct in M1 vs M0 macrophages. FRET shows the sensitized emission (magenta in merge), mScarlet-I shows the acceptor fluorescence (orange), mCitrine shows the donor emission (green). FRET efficiency shows the ratio of sensitized emission over the donor emission in glow lookup table. Scale bars: 5 μm.

Finally, we used the FRET probes to determine the processing of CgA in human macrophages polarized to proinflammatory and anti-inflammatory phenotypes. Our results show that, compared to nonactivated and anti-inflammatory macrophages, the proinflammatory macrophages have lower FRET efficiency both for the PST and CST probes (Fig. 6). This aligns with increased proteolytic digestion of the FRET probes, indicating that macrophages show differential CgA processing depending on their inflammatory phenotype. Importantly, we observed this effect both for live cells and cells that were fixed and permeabilized, indicating that it was not due to differences in luminal pH or ion concentrations between the macrophage phenotypes (Supplementary Fig. S6 [60]).

## Discussion

CgA and CgA-derived peptides are involved in many pathological conditions, and it is increasingly clear that they are key regulators of the nervous, endocrine, and immune systems [22, 24, 36, 38]. However, the mechanism of how CgA and its derived peptides modulate the immune response remains unclear. There are 3 nonmutually exclusive possibilities of how macrophages could be involved in this process:

First, macrophages could be a source of CgA themselves. This possibility is supported by the finding that, although CgA is produced mainly by chromaffin and other neuroendocrine cells, Western blot analysis detected the presence of CgA and CST in intraperitoneal macrophages of wild-type mice [7, 77]. Moreover, the metabolic and inflammatory phenotypes of CST-KO mice could be reversed with a bone marrow transplant from wild-type mice, whereas these phenotypes could also be transferred by bone marrow transplant from CST-KO mice to wild-type mice [7]. However, we did not detect CgA gene expression by RT-qPCR analyses in murine bone marrow–derived and human monocyte–derived macrophages. Nevertheless, we did observe some CgA expression in murine bone marrow. Since we could not detect CgA expression in monocytes and neutrophils, CgA might be expressed in other bone marrow–derived cells that we did not assess. Alternatively, or additionally, CgA could be expressed in neuronal cells that innervate the bone marrow [78].

The second possibility is that macrophages ingest CgA and process it into CST and PST, as this might also explain why CgA and CST are detected in murine peritoneal macrophages. However, CgA was not detected in human monocytes or monocyte-derived macrophages, whereas CST is [7, 77]. Moreover, it does not seem to explain why the phenotype of CST-KO and wild-type mice can be exchanged by bone marrow transfer [7]. Nevertheless, we found that inflammatory macrophage activation increases the cleavage of the FRET probes both for N- and C-terminal cleavage sites of CST and PST. This differential processing was expected as cathepsin L expression is also macrophage phenotype dependent [79]. These findings suggest that inflammatory activation of macrophages results in increased conversion of CgA to CST and PST.

Third, macrophages could be affected by CST and PST. If this already occurs in early development, this could also explain the transfer of the phenotypes between CST-KO mice and wild-type mice by bone marrow transplant [7]. This could also explain why the depletion of macrophages using chlodronate restores the proinflammatory phenotype of the CST-KO mice [7]. However, this cannot explain the detection of CgA and CST in intraperitoneal macrophages by Western blot [7, 77]. Supporting this possibility is the finding that treatment of cultured peritoneal isolated macrophages derived from wild-type and *CHGA*^−/−^ mice with PST resulted in the differentiation of these macrophages toward a proinflammatory phenotype [37], which correlates with increased levels of PST in several diseases [22, 24, 38]. Moreover, treatment of murine macrophages with CST resulted in a small but statistically significant increase in the production of IL-10 and a decrease of TNF-α, IL-1β, CCL-2, CCL-3, and CXCL-1 [7]. Our study showed a minor but consistent and significant decrease in IL-6 production when human monocyte–derived macrophages were treated with CST or PST alone. PST also reduced COX2 expression, whereas CST reduced fat storage of the macrophages. Thus, CST and PST both seem to have small but consistent effects, and these compounds reduce specific inflammatory parameters (Table 2). Therefore, these findings might at least partly explain the anti-inflammatory roles of CST [38]. Indeed, it has already been shown that treatment of murine macrophages in vivo and in vitro reduces the production of proinflammatory cytokines, including IL-6 [80, 81]. However, the effects are only small and unlikely to completely explain the anti-inflammatory effects of CST. Moreover, our findings cannot explain the proinflammatory roles of PST [22] as it, for example, is known to induce IL-6 in adipose tissue macrophages [32]. Thus, our findings are difficult to reconcile with the opposing effects of CST and PST in inflammation.

**Table 2.** Summary of significant effects of catestatin and pancreastatin

Parameter	CST	PST
IL-6	↓ at 6 h (*P* = 0.0012)	↓ at 6 h (*P* = .0235)
COX-2	—	↓ at 6 h (*P* = .0237)
BODIPY 493/503	↑ at 7 d (*P* = .0352)	—
Intracellular lactate	↓ at 3 h (*P* = .0463)	—
CST-FITC uptake	M0 > M1 (*P* = .0045)	—
FRET ratio, N-terminal	M0 > M1 (*P* = .0118) M1 < M2 (*P* = .0198)	M0 > M1 (*P* = .0004) M1 < M2 (*P* = .0308)
FRET ratio, C-terminal	M0 > M1 (*P* = .0079)	M0 > M1 (*P* = .0031)

Abbreviations: COX-2, cyclooxygenase-2; CST, catestatin; FITC, fluorescein isothiocyanate; FRET, Förster resonance energy transfer; IL-6, interleukin-6; PST, pancreastatin.

IL-6 is a multifaceted cytokine that can stimulate and reduce inflammation [82]. IL-6 regulates acute-phase protein production, immune responses, inflammation, and immune metabolism. Metabolic changes like obesity can increase IL-6 levels and promote immune tolerance, revealing a complex link between inflammation and metabolism [83]. Moreover, IL-6 is involved in regulating insulin action, and elevated levels of IL-6 in obesity are a predictive factor for developing type 2 diabetes [84, 85]. In addition, IL-6 regulates cell proliferation and survival, particularly in liver and immune cells [86].

We also found that CST slightly but significantly increased fatty acid storage by macrophages, as was already described for hepatocytes (HepG2) and adipocytes (3T3-L1 cells) [87]. An increase in macrophage fatty acid storage typically indicates a shift toward an anti-inflammatory phenotype. Enhanced fatty acid storage can result in more β-oxidation, which results in a more anti-inflammatory state by modulating the energy balance of macrophages [67].

Thus, the reduction of IL-6 by CST, as well as the increased lipid uptake, support previous data about the anti-inflammatory effect of CST. However, the reduction of IL-6 and COX2 by PST does not support the proinflammatory role of PST. COX2 plays an important role in macrophage inflammatory responses. It catalyzes the rate-limiting step in synthesizing prostaglandins from arachidonic acid. COX2 is quickly expressed on inflammation and increases prostaglandins [88]. Prostaglandins can modulate the inflammatory response toward the resolution or the stimulation of inflammation [89]. Interestingly, COX2 can promote CgA expression in a prostaglandin E2–dependent manner, which directs the neuroendocrine cell-specific expression of this protein in a rat pheochromocytoma (PC12) cell line [90]. The reduction of COX2 by PST might suggest a regulatory feedback loop that could influence the immune response.

In summary, we found that macrophages do not produce CgA. However, they can convert CgA both to PST and CST, and this conversion is increased on inflammatory activation. Moreover, PST and CST both exert small but significant anti-inflammatory effects on human macrophages that are difficult to reconcile with the proinflammatory effect of PST.

## Data Availability

Original data generated and analyzed during this study are included in this published article or in the data repositories listed in the “References” [60]. Plasmids have been deposited at Addgene.

## Abbreviations

BMDMs: bone marrow–derived macrophages
cDNA: complementary DNA
*Chga* ^−/−^: CgA-knockout mice
CgA: chromogranin A
COX-2: cyclooxygenase-2
CST: catestatin
DAPI: 4′,6-diamidino-2-phenylindole dihydrochloride
FBS: fetal bovine serum
FITC: fluorescein isothiocyanate
FRET: Förster resonance energy transfer
GFP: green fluorescent protein
IFN-γ: interferon-γ
IL: interleukin
iNOS: inducible nitric oxide synthase
KO: knockout
LPS: lipopolysaccharide
M-CSF: macrophage colony-stimulating factor
Mcp: monocyte chemoattractant protein
MTT: 3-(4,5-dimethyl-2-thiazolyl)-2,5-diphenyl-2H-tetrazolium bromide
PBS: phosphate-buffered saline
PCs: prohormone convertases
PFA: paraformaldehyde
PST: pancreastatin
qPCR: quantitative polymerase chain reaction
RRID: Research Resource Identifier
RT: reverse-transcription
SDH: succinate dehydrogenase
TNF-α: tumor necrosis factor-α

## References

[1] Winkler H, Fischer-Colbrie R. The chromogranins A and B: the first 25 years and future perspectives. Neuroscience. 1992;49(3):497‐528. 1501763 10.1016/0306-4522(92)90222-N PMC7131462

[2] Montero-Hadjadje M, Vaingankar S, Elias S, Tostivint H, Mahata SK, Anouar Y. Chromogranins A and B and secretogranin II: evolutionary and functional aspects. Acta Physiologica. 2008;192(2):309‐324. 18005393 10.1111/j.1748-1716.2007.01806.x

[3] Hosaka M, Watanabe T, Sakai Y, Uchiyama Y, Takeuchi T. Identification of a chromogranin A domain that mediates binding to secretogranin III and targeting to secretory granules in pituitary cells and pancreatic β-cells. Mol Biol Cell. 2002;13(10):3388‐3399. 12388744 10.1091/mbc.02-03-0040 PMC129953

[4] Liu MA, Shahabi S, Jati S, et al Gut microbial DNA and immune checkpoint gene Vsig4/CRIg are key antagonistic players in healthy aging and age-associated development of hypertension and diabetes. Front Endocrinol (Lausanne). 2022;13:1037465. 36440192 10.3389/fendo.2022.1037465 PMC9691654

[5] Muntjewerff EM, Tang K, Lutter L, et al Chromogranin A regulates gut permeability via the antagonistic actions of its proteolytic peptides. Acta Physiologica. 2021;232(2):e13655. 33783968 10.1111/apha.13655 PMC8341099

[6] Bandyopadhyay G, Tang K, Webster NJG, van den Bogaart G, Mahata SK. Catestatin induces glycogenesis by stimulating the phosphoinositide 3-kinase-AKT pathway. Acta Physiologica. 2022;235(1):e13775. 34985191 10.1111/apha.13775 PMC10754386

[7] Ying W, Tang K, Avolio E, et al Immunosuppression of macrophages underlies the cardioprotective effects of CST (catestatin). Hypertension. 2021;77(5):1670‐1682. 33826401 10.1161/HYPERTENSIONAHA.120.16809 PMC8116433

[8] Sahu BS, Mahata S, Bandyopadhyay K, et al Catestatin regulates vesicular quanta through modulation of cholinergic and peptidergic (PACAPergic) stimulation in PC12 cells. Cell Tissue Res. 2019;376(1):51‐70. 30467710 10.1007/s00441-018-2956-1

[9] Mahapatra NR, O’Connor DT, Vaingankar SM, et al Hypertension from targeted ablation of chromogranin A can be rescued by the human ortholog. J Clin Invest. 2005;115(7):1942‐1952. 16007257 10.1172/JCI24354 PMC1159140

[10] Gayen JR, Saberi M, Schenk S, et al A novel pathway of insulin sensitivity in chromogranin A null mice: a CRUCIAL ROLE FOR PANCREASTATIN IN GLUCOSE HOMEOSTASIS *. J Biol Chem. 2009;284(42):28498‐28509. 19706599 10.1074/jbc.M109.020636 PMC2781393

[11] Lee SH, Jo JH, Kim YJ, et al Plasma chromogranin A as a prognostic marker in pancreatic ductal adenocarcinoma. Pancreas. 2019;48(5):662‐669. 31091213 10.1097/MPA.0000000000001319

[12] Sobol RE . Elevated Serum chromogranin A concentrations in small-cell lung carcinoma. Ann Intern Med. 1986;105(5):698. 3021037 10.7326/0003-4819-105-5-698

[13] O’Connor DT, Deftos LJ. Secretion of chromogranin A by peptide-producing endocrine neoplasms. N Engl J Med. 1986;314(18):1145‐1151. 3007986 10.1056/NEJM198605013141803

[14] O’Connor DT . Plasma chromogranin A. Initial studies in human hypertension. Hypertension. 1985;7(3_pt_2):I76-9. 3997234 10.1161/01.hyp.7.3_pt_2.i76

[15] Pieroni M, Corti A, Tota B, et al Myocardial production of chromogranin A in human heart: a new regulatory peptide of cardiac function. Eur Heart J. 2007;28(9):1117‐1127. 17389654 10.1093/eurheartj/ehm022

[16] O’Connor DT . Chromogranin A: implications for hypertension. J Hypertens Suppl. 1984;2(3):S147‐S150. 6599663

[17] Hsiao RJ, Mezger MS, O’Connor DT. Chromogranin A in uremia: progressive retention of immunoreactive fragments. Kidney Int. 1990;37(3):955‐964. 2313983 10.1038/ki.1990.71

[18] Salem RM, Cadman PE, Chen Y, et al Chromogranin A polymorphisms are associated with hypertensive renal disease. J Am Soc Nephrol. 2008;19(3):600‐614. 18235090 10.1681/ASN.2007070754 PMC2391050

[19] Di Comite G, Rossi CM, Marinosci A, et al Circulating chromogranin A reveals extra-articular involvement in patients with rheumatoid arthritis and curbs TNF- -elicited endothelial activation. J Leukoc Biol. 2008;85(1):81-7. 18832606 10.1189/jlb.0608358

[20] Sciola V, Massironi S, Conte D, et al Plasma chromogranin a in patients with inflammatory bowel disease. Inflamm Bowel Dis. 2009;15(6):867‐871. 19090560 10.1002/ibd.20851

[21] Zhernakova A, Kurilshikov A, Bonder MJ, et al Population-based metagenomics analysis reveals markers for gut microbiome composition and diversity. Science (1979). 2016;352(6285):565‐569. 10.1126/science.aad3369 PMC5240844 27126040

[22] Ioannidis M, Mahata SK, van den Bogaart G. The immunomodulatory functions of chromogranin A-derived peptide pancreastatin. Peptides (NY). 2022;158:170893. 10.1016/j.peptides.2022.170893 PMC10760928 36244579

[23] Bandyopadhyay GK, Mahata SK. Chromogranin A regulation of obesity and peripheral insulin sensitivity. Front Endocrinol (Lausanne). 2017;8:20. 28228748 10.3389/fendo.2017.00020 PMC5296320

[24] Mahata SK, Corti A. Chromogranin A and its fragments in cardiovascular, immunometabolic, and cancer regulation. Ann N Y Acad Sci. 2019;1455(1):34‐58. 31588572 10.1111/nyas.14249 PMC6899468

[25] Konecki DS, Benedum UM, Gerdes HH, Huttner WB. The primary structure of human chromogranin A and pancreastatin. J Biol Chem. 1987;262(35):17026‐17030. 2445752

[26] Mosley CA, Taupenot L, Biswas N, et al Biogenesis of the secretory granule: chromogranin A coiled-coil structure results in unusual physical properties and suggests a mechanism for granule core condensation. Biochemistry. 2007;46(38):10999‐11012. 17718510 10.1021/bi700704r

[27] Aardal S, Helle KB. The vasoinhibitory activity of bovine chromogranin A fragment (vasostatin) and its independence of extracellular calcium in isolated segments of human blood vessels. Regul Pept. 1992;41(1):9‐18. 1455014 10.1016/0167-0115(92)90509-s

[28] Curry WJ, Shaw C, Johnston CF, Thim L, Buchanan KD. Isolation and primary structure of a novel chromogranin A-derived peptide, WE-14, from a human midgut carcinoid tumour. FEBS Lett. 1992;301(3):319‐321. 1577173 10.1016/0014-5793(92)80266-j

[29] Koshimizu H, Cawley NX, Kim T, Yergey AL, Loh YP. Serpinin: a novel chromogranin A-derived, secreted peptide up-regulates protease nexin-1 expression and granule biogenesis in endocrine cells. Mol Endocrinol. 2011;25(5):732‐744. 21436258 10.1210/me.2010-0124 PMC3082324

[30] Mahata SK, O’Connor DT, Mahata M, et al Novel autocrine feedback control of catecholamine release. A discrete chromogranin a fragment is a noncompetitive nicotinic cholinergic antagonist. J Clin Invest. 1997;100(6):1623‐1633. 9294131 10.1172/JCI119686 PMC508344

[31] Tatemoto K, Efendić S, Mutt V, Makk G, Feistner GJ, Barchas JD. Pancreastatin, a novel pancreatic peptide that inhibits insulin secretion. Nature. 1986;324(6096):476‐478. 3537810 10.1038/324476a0

[32] Bandyopadhyay GK, Lu M, Avolio E, et al Pancreastatin-dependent inflammatory signaling mediates obesity-induced insulin resistance. Diabetes. 2015;64(1):104‐116. 25048197 10.2337/db13-1747

[33] Ying W, Mahata S, Bandyopadhyay GK, et al Catestatin inhibits obesity-induced macrophage infiltration and inflammation in the liver and suppresses hepatic glucose production, leading to improved insulin sensitivity. Diabetes. 2018;67(5):841‐848. 29432123 10.2337/db17-0788 PMC6463753

[34] Videen JS, Mezger MS, Chang YM, O’Connor DT. Calcium and catecholamine interactions with adrenal chromogranins. Comparison of driving forces in binding and aggregation. J Biol Chem. 1992;267(5):3066‐3073. 1737762

[35] Mahata SK, Kiranmayi M, Mahapatra NR. Catestatin: a master regulator of cardiovascular functions. Curr Med Chem. 2018;25(11):1352‐1374. 28443506 10.2174/0929867324666170425100416

[36] Mahata SK, Mahata M, Fung MM, O’Connor DT. Catestatin: a multifunctional peptide from chromogranin A. Regul Pept. 2010;162(1–3):33‐43. 20116404 10.1016/j.regpep.2010.01.006 PMC2866790

[37] Eissa N, Elgazzar O, Hussein H, Hendy GN, Bernstein CN, Ghia JE. Pancreastatin reduces alternatively activated macrophages, disrupts the epithelial homeostasis and aggravates colonic inflammation. A descriptive analysis. Biomedicines. 2021;9(2):134. 33535452 10.3390/biomedicines9020134 PMC7912769

[38] Muntjewerff EM, Dunkel G, Nicolasen MJT, Mahata SK, van den Bogaart G. Catestatin as a target for treatment of inflammatory diseases. Front Immunol. 2018;9:2199. 30337922 10.3389/fimmu.2018.02199 PMC6180191

[39] O’Connor DT, Cadman PE, Smiley C, et al Pancreastatin: multiple actions on human intermediary metabolism in vivo, variation in disease, and naturally occurring functional genetic polymorphism. J Clin Endocrinol Metab. 2005;90(9):5414‐5425. 15956083 10.1210/jc.2005-0408

[40] O’Connor DT, Kailasam MT, Kennedy BP, Ziegler MG, Yanaihara N, Parmer RJ. Early decline in the catecholamine release-inhibitory peptide catestatin in humans at genetic risk of hypertension. J Hypertens. 2002;20(7):1335‐1345. 12131530 10.1097/00004872-200207000-00020

[41] Kennedy BP, Mahata SK, O’Connor DT, Ziegler MG. Mechanism of cardiovascular actions of the chromogranin A fragment catestatin in vivo. Peptides (NY). 1998;19(7):1241‐1248. 10.1016/s0196-9781(98)00086-2 9786174

[42] Muntjewerff EM, Christoffersson G, Mahata SK, van den Bogaart G. Putative regulation of macrophage-mediated inflammation by catestatin. Trends Immunol. 2022;43(1):41‐50. 34844850 10.1016/j.it.2021.11.002 PMC10843896

[43] Lee JC, Taylor C V, Gaucher SP, et al Primary sequence characterization of catestatin intermediates and peptides defines proteolytic cleavage sites utilized for converting chromogranin A into active catestatin secreted from neuroendocrine chromaffin cells. Biochemistry. 2003;42(23):6938‐6946. 12795588 10.1021/bi0300433

[44] Taylor C V, Taupenot L, Mahata SK, et al Formation of the catecholamine release-inhibitory peptide catestatin from chromogranin A. J Biol Chem. 2000;275(30):22905‐22915. 10781584 10.1074/jbc.M001232200

[45] Metz-Boutigue M, Garcia-Sablone P, Hogue-Angeletti R, Aunis D. Intracellular and extracellular processing of chromogranin A. Eur J Biochem. 1993;217(1):247‐257. 8223562 10.1111/j.1432-1033.1993.tb18240.x

[46] Biswas N, Rodriguez-Flores JL, Courel M, et al Cathepsin L colocalizes with chromogranin A in chromaffin vesicles to generate active peptides. Endocrinology. 2009;150(8):3547‐3557. 19372204 10.1210/en.2008-1613 PMC2717865

[47] Crippa L, Bianco M, Colombo B, et al A new chromogranin A–dependent angiogenic switch activated by thrombin. Blood. 2013;121(2):392‐402. 23190532 10.1182/blood-2012-05-430314 PMC3544118

[48] Biswas N, Vaingankar SM, Mahata M, et al Proteolytic cleavage of human chromogranin A containing naturally occurring catestatin variants: differential processing at catestatin region by plasmin. Endocrinology. 2008;149(2):749‐757. 17991725 10.1210/en.2007-0838 PMC2219303

[49] Parmer RJ, Mahata M, Gong Y, et al Processing of chromogranin A by plasmin provides a novel mechanism for regulating catecholamine secretion. J Clin Invest. 2000;106(7):907‐915. 11018079 10.1172/JCI7394 PMC381423

[50] Rouillé Y, Bianchi M, Irminger JC, Halban PA. Role of the prohormone convertase PC2 in the processing of proglucagon to glucagon. FEBS Lett. 1997;413(1):119‐123. 9287128 10.1016/s0014-5793(97)00892-2

[51] Smeekens SP, Montag AG, Thomas G, et al Proinsulin processing by the subtilisin-related proprotein convertases furin, PC2, and PC3. Proc Natl Acad Sci U S A. 1992;89(18):8822‐8826. 1528899 10.1073/pnas.89.18.8822 PMC50013

[52] Gomes CP, Fernandes DE, Casimiro F, et al Cathepsin L in COVID-19: from pharmacological evidences to genetics. Front Cell Infect Microbiol. 2020;10:589505. 33364201 10.3389/fcimb.2020.589505 PMC7753008

[53] Mezouar S, Frère C, Darbousset R, et al Role of platelets in cancer and cancer-associated thrombosis: experimental and clinical evidences. Thromb Res. 2016;139:65‐76. 26916298 10.1016/j.thromres.2016.01.006

[54] Cesarman-Maus G, Hajjar KA. Molecular mechanisms of fibrinolysis. Br J Haematol. 2005;129(3):307‐321. 15842654 10.1111/j.1365-2141.2005.05444.x

[55] Maassen S, Coenen B, Ioannidis M, et al Itaconate promotes a wound resolving phenotype in pro-inflammatory macrophages. Redox Biol. 2023;59:102591. 36574745 10.1016/j.redox.2022.102591 PMC9800195

[56] Paardekooper LM, Dingjan I, Linders PTA, et al Human monocyte-derived dendritic cells produce millimolar concentrations of ROS in phagosomes per second. Front Immunol. 2019;10:1216. 31191556 10.3389/fimmu.2019.01216 PMC6548834

[57] Bohman S, Andersson A, King A. No differences in efficacy between noncultured and cultured islets in reducing hyperglycemia in a nonvascularized islet graft model. Diabetes Technol Ther. 2006;8(5):536‐545. 17037968 10.1089/dia.2006.8.536

[58] Livak KJ, Schmittgen TD. Analysis of relative gene expression data using real-time quantitative PCR and the 2−ΔΔCT method. Methods. 2001;25(4):402‐408. 11846609 10.1006/meth.2001.1262

[59] Stempels FC, Jiang M, Warner HM, et al Giant worm-shaped ESCRT scaffolds surround actin-independent integrin clusters. J Cell Biol. 2023;222(7):e202205130. 37200023 10.1083/jcb.202205130 PMC10200693

[60] Ioannidis M, van Dijk H, Muntjewerff EM, et al Data set: “Inflammation Promotes Proteolytic Processing of the Prohormone Chromogranin A by Macrophages”. Zenodo. 2025. https://zenodo.org/records/15276276 10.1210/jendso/bvaf090 PMC12127132 40458085

[61] Wollam J, Mahata S, Riopel M, et al Chromogranin A regulates vesicle storage and mitochondrial dynamics to influence insulin secretion. Cell Tissue Res. 2017;368(3):487‐501. 28220294 10.1007/s00441-017-2580-5 PMC10843982

[62] Corti A, Marcucci F, Bachetti T. Circulating chromogranin A and its fragments as diagnostic and prognostic disease markers. Pflugers Arch. 2018;470(1):199‐210. 29018988 10.1007/s00424-017-2030-y

[63] Tateishi K, Kitayama N, Matsuoka Y, Funakoshi A. Comparison of chromogranin a and pancreastatin levels in plasma of patients with pancreatic islet cell tumor. Life Sci. 1995;57(9):889‐895. 7630318 10.1016/0024-3205(95)02022-b

[64] Rustagi S, Warner RRP, Divino CM. Serum pancreastatin: the next predictive neuroendocrine tumor marker. J Surg Oncol. 2013;108(2):126‐128. 23775817 10.1002/jso.23359

[65] Li Y, Oosting M, Deelen P, et al Inter-individual variability and genetic influences on cytokine responses to bacteria and fungi. Nat Med. 2016;22(8):952‐960. 27376574 10.1038/nm.4139 PMC5084084

[66] Nørregaard R, Kwon TH, Frøkiær J. Physiology and pathophysiology of cyclooxygenase-2 and prostaglandin E2 in the kidney. Kidney Res Clin Pract. 2015;34(4):194‐200. 26779421 10.1016/j.krcp.2015.10.004 PMC4688592

[67] Viola A, Munari F, Sánchez-Rodríguez R, Scolaro T, Castegna A. The metabolic signature of macrophage responses. Front Immunol. 2019;10:1462. 31333642 10.3389/fimmu.2019.01462 PMC6618143

[68] Williams NC, O’Neill LAJ. A role for the krebs cycle intermediate citrate in metabolic reprogramming in innate immunity and inflammation. Front Immunol. 2018;9:141. 29459863 10.3389/fimmu.2018.00141 PMC5807345

[69] Lachmandas E, Boutens L, Ratter JM, et al Microbial stimulation of different toll-like receptor signalling pathways induces diverse metabolic programmes in human monocytes. Nat Microbiol. 2016;2(3):16246. 27991883 10.1038/nmicrobiol.2016.246

[70] Li Y, He Y, Miao K, Zheng Y, Deng C, Liu TM. Imaging of macrophage mitochondria dynamics *in vivo* reveals cellular activation phenotype for diagnosis. Theranostics. 2020;10(7):2897‐2917. 32194843 10.7150/thno.40495 PMC7053213

[71] Sánchez-Margalet V, González-Yanes C. Pancreastatin inhibits insulin action in rat adipocytes. Am J Physiol Endocrinol Metab. 1998;275(6):E1055‐E1060. 10.1152/ajpendo.1998.275.6.E1055 9843749

[72] Chen S, Saeed AFUH, Liu Q, et al Macrophages in immunoregulation and therapeutics. Signal Transduct Target Ther. 2023;8(1):207. 37211559 10.1038/s41392-023-01452-1 PMC10200802

[73] Jumper J, Evans R, Pritzel A, et al Highly accurate protein structure prediction with AlphaFold. Nature. 2021;596(7873):583‐589. 34265844 10.1038/s41586-021-03819-2 PMC8371605

[74] Stettler H, Beuret N, Prescianotto-Baschong C, Fayard B, Taupenot L, Spiess M. Determinants for chromogranin A sorting into the regulated secretory pathway are also sufficient to generate granule-like structures in non-endocrine cells. Biochem J. 2009;418(1):81‐91. 18973469 10.1042/BJ20071382

[75] Taupenot L, Harper KL, Mahapatra NR, Parmer RJ, Mahata SK, O’Connor DT. Identification of a novel sorting determinant for the regulated pathway in the secretory protein chromogranin A. J Cell Sci. 2002;115(24):4827‐4841. 12432071 10.1242/jcs.00140

[76] Jares-Erijman EA, Jovin TM. FRET imaging. Nat Biotechnol. 2003;21(11):1387‐1395. 14595367 10.1038/nbt896

[77] Kojima M, Ozawa N, Mori Y, et al Catestatin prevents macrophage-driven atherosclerosis but not arterial injury–induced neointimal hyperplasia. Thromb Haemost. 2018;118(01):182‐194. 29304538 10.1160/TH17-05-0349

[78] Maryanovich M, Takeishi S, Frenette PS. Neural regulation of bone and bone marrow. Cold Spring Harb Perspect Med. 2018;8(9):a031344. 29500307 10.1101/cshperspect.a031344 PMC6119651

[79] Wang F, Baverel V, Chaumonnot K, et al The endoplasmic reticulum stress protein GRP94 modulates cathepsin L activity in M2 macrophages in conditions of obesity-associated inflammation and contributes to their pro-inflammatory profile. Int J Obes. 2024;48(6):830‐840. 10.1038/s41366-024-01478-7 38351251

[80] Rabbi MF, Eissa N, Munyaka PM, et al Reactivation of intestinal inflammation is suppressed by catestatin in a murine model of colitis via M1 macrophages and not the gut Microbiota. Front Immunol. 2017;8:985. 28871257 10.3389/fimmu.2017.00985 PMC5566981

[81] Rabbi MF, Labis B, Metz-Boutigue MH, Bernstein CN, Ghia JE. Catestatin decreases macrophage function in two mouse models of experimental colitis. Biochem Pharmacol. 2014;89(3):386‐398. 24637240 10.1016/j.bcp.2014.03.003

[82] Hunter CA, Jones SA. Erratum: corrigendum: IL-6 as a keystone cytokine in health and disease. Nat Immunol. 2017;18(11):1271‐1271. 10.1038/ni1117-1271b 29044237

[83] Hotamisligil GS . Inflammation, metaflammation and immunometabolic disorders. Nature. 2017;542(7640):177‐185. 28179656 10.1038/nature21363

[84] Spranger J, Kroke A, Möhlig M, et al Inflammatory cytokines and the risk to develop type 2 diabetes. Diabetes. 2003;52(3):812‐817. 12606524 10.2337/diabetes.52.3.812

[85] Herder C, Haastert B, Müller-Scholze S, et al Association of systemic chemokine concentrations with impaired glucose tolerance and type 2 diabetes. Diabetes. 2005;54(suppl_2):S11‐S17. 16306328 10.2337/diabetes.54.suppl_2.s11

[86] Kamimura D, Ishihara K, Hirano T. IL-6 signal transduction and its physiological roles: the signal orchestration model. Rev Physiol Biochem Pharmacol. 2003;149:1‐38. 12687404 10.1007/s10254-003-0012-2

[87] Bandyopadhyay GK, Vu CU, Gentile S, et al Catestatin (chromogranin A352–372) and novel effects on mobilization of fat from adipose tissue through regulation of adrenergic and leptin signaling. J Biol Chem. 2012;287(27):23141‐23151. 22535963 10.1074/jbc.M111.335877 PMC3391131

[88] Smith WL, DeWitt DL, Garavito RM. Cyclooxygenases: structural, cellular, and molecular biology. Annu Rev Biochem. 2000;69(1):145‐182. 10966456 10.1146/annurev.biochem.69.1.145

[89] Takayama K, García-Cardeña G, Sukhova GK, Comander J, Gimbrone MA, Libby P. Prostaglandin E2 suppresses chemokine production in human macrophages through the EP4 receptor. J Biol Chem. 2002;277(46):44147‐44154. 12215436 10.1074/jbc.M204810200

[90] Connolly R, Gates D, Loh N, et al Cox-2 promotes chromogranin A expression and bioactivity: evidence for a prostaglandin E2-dependent mechanism and the involvement of a proximal cyclic adenosine 5′-monophosphate-responsive element. Endocrinology. 2007;148(9):4310‐4317. 17540723 10.1210/en.2007-0167

